# Isolation and characterization of bacteriophages with lytic activity against common bacterial pathogens

**DOI:** 10.14202/vetworld.2017.973-978

**Published:** 2017-08-23

**Authors:** R. K. Shende, S. D. Hirpurkar, C. Sannat, Nidhi Rawat, Vinay Pandey

**Affiliations:** 1 Department of Veterinary Microbiology, College of Veterinary Science & Animal Husbandry, Anjora Chhattisgarh Kamdhenu Vishwavidyalaya, Durg, Chhattisgarh, India; 2 Department of Animal Nutrition, College of Veterinary Science & Animal Husbandry, Anjora, Chhattisgarh Kamdhenu Vishwavidyalaya, Durg, Chhattisgarh, India

**Keywords:** *Bacillus subtilis*, bacteriophage, *Escherichia coli*, host range, sewage

## Abstract

**Aim:**

Present investigation was conducted to isolate and characterize bacteriophages with lytic activity against common bacterial pathogens.

**Materials and Methods:**

A total of 60 samples of animal waste disposal from cattle (42) and buffalo (18) farms were collected from three different strata, i.e., top, mid, and bottom of collection tank. Samples were primarily subjected to rapid detection methods, and then isolation of phage was done by double agar layer method using *Bacillus subtilis* (BsH) and *Escherichia coli* (EH) as host system. Phages were characterized on the basis of plaque morphology, temperature, pH susceptibility, and host range.

**Results:**

Recovery of phages was higher from dairy cattle farm waste (78.57%) as compared to buffalo farm waste (72.22%) and bottom layer of tank showed maximum recovery. *Bacillus subtilis* (91%) supported the growth of more phages as compared to *E. coli* (9%). Three different phage morphotypes were observed each against *Bacillus subtilis* (BsHR_1_, BsHR_2_, and BsHR_3_) and *E. coli* (EHR1, EHR_2_, and EHR_3_). Mean phage titer of above six phage isolates ranged between 3×10^10^ and 5×10^12^ plaque forming units/ml. Viability of phages was by, and large unaffected at 70°C within 2-3 min, and phage isolates were completely inactivated below pH 3 and above 11. Coliphage EHR_1_ had widest host range followed by BsHR_1_ and BsHR_2_ while EHR_2_, EHR_3_, and BsHR_3_ had low lytic activity.

**Conclusion:**

It could be concluded from the present study that the *Bacillus* and *Coli* phage has wide host range and thus exhibits the potential to be used as drug substitute tool against common bacterial pathogens.

## Introduction

Bacteriophages are viruses which have the ability to multiply only in bacterial cells, and they are detectable almost everywhere where live bacteria exist [1]. The environment populated by bacterial hosts such as soil, sewage, and animal secretions are unique source of all types of phages, offering the possibility to isolate them for therapeutic purposes [2]. In current scenario, serious medical and social problem results from the increasing antibiotic resistance of bacterial strains [3]. At the same time, pharmaceutical industries are withdrawing from research and development on new antibiotics due to unprofitability of the venture and the risks of development of resistance in bacteria [4], which has generated interest in alternatives over conventional and current system of microbial control. Lytic phages are the possible replacement for antibiotics to treat bacterial infections not responding to conventional antibiotic therapy [5].

The application of phages to control a certain bacterial pathogen is complicated by the high degrees of phenotypic diversity within populations of both phages and bacteria [6]. Consequently, individual strains of a pathogen may be more or less susceptible or even resistant to different co-occurring phages. Before therapeutic application, it is necessary to understand in detail the phage and host interaction, which is affected by both biological and physical factors [7]. The biological aspect is related to bacterial resistance, whereas temperature and pH are the main physical factors affecting the phage adsorption and bacterial growth.

Therefore, the present investigation was conducted to isolate and characterize lytic bacteriophages from the dairy farm waste using *Escherichia coli* and *Bacillus subtilis* as host system and to assess their *in vitro* susceptibility in common bacterial pathogens isolated from clinical cases.

## Materials and Methods

### Ethical approval

Ethical approval was not necessary to pursue this research work.

### Location of study

The present study was conducted at the Department of Veterinary Microbiology, College of Veterinary Science and Animal Husbandry, Anjora, Durg, Chhattisgarh, India.

### Host bacteria

*B. subtilis* and *E. coli* obtained from sewage were used as primary hosts for the isolation of phages.

### Collection and processing of waste water samples

The samples of animal waste disposal constituting various body excretions of cows (42 nos.) and buffaloes (18 nos.) were collected in sufficient amount from three different strata, i.e. top, mid (15 cm deep from top), and bottom (45 cm deep from top) of collection tank. For this purpose, 30 cm long, sterile disposable pipette, pipette bulb, and conical flasks were used.

Samples were processed as per the method given by Jothikumar *et al*. [8] with slight modifications. Samples were spun gently in a homogenizer for 3 h continuously and then centrifuged at 3000 rpm for 20 min. The supernatant was collected and recentrifuged at 5000 rpm for 20 min in refrigerated centrifuge (Remi C-24). Then, supernatant was filtered through a 0.45 µm millipore syringe filters, and filtrate was tested for the presence of lytic activity against *B. subtilis* and *E. coli*.

### Isolation of bacteriophage

Turbidity reduction and streak plate technique were used as preliminary methods for screening of lytic activity of phages. Turbidity reduction method was performed as per protocol of Harrigan and McCance [9] with slight modifications. 1 ml chloroform was added in 5 ml of processed sewage sample, then mixed and centrifuged at 3000 rpm for 20 min. 1 ml supernatant was transferred in 5 ml broth culture of bacteria. Whereas in streak plate method, processed samples were inoculated over surface of bacterial lawn on nutrient agar plates. Next day plates were examined for the presence of plaques or any lytic activity on bacterial lawn. The test was repeated thrice before the sample is adjudged as negative.

On the basis of clear plaque morphology, samples were selected for phage lysate preparation by more sensitive double agar layer (DAL) method as described by Adams [10]. In a sterile vial, 500 µl of filtrate was mixed with 500 µl of 6 h old bacterial culture, and 2.4 g/L MgCl_2_ solution was added to enhance the adsorption of phage over bacterial surface. Then, it was kept on a shaker in gentle speed for 20 min and then added to test tube containing 2.5 ml of molten soft agar held at 45°C in a water bath. The mixture was swirled, poured onto lactose agar basal plates, allowed to solidify and then incubated at 37°C up to 48 h. Plates were observed at interval of 6, 12, 24, and 48 h for the development of plaques. Phage lysate was purified by two consecutive single plaque isolation cycle and propagated on the corresponding bacterial host strain. Different phage lysates were designated by the laboratory identification number as BsHR for *B. subtilis* phages and EHR for *E. coli* phages.

### Characterization of phages

#### Host range determination

Freshly recovered phages were tested against pathogenic bacteria, namely, *Staphylococcus aureus*, *Salmonella* spp., *Pseudomonas aeruginosa*, *E. coli*, *Proteus* spp., and *Klebsiella* spp. isolated from different clinical samples.

#### Plaque morphology

Morphology of plaque was recorded according to their size, edge, and boundaries [11] and was noted as small (under 2 mm), medium (2 mm), and large (larger than 2 mm)/clear or diffused type plaques.

#### Effect of temperature and pH

Viability of phage was tested at 70°C temperature when exposed for 1, 2, and 3 min. Phage viability before and after heat treatment was adjudged by DAL method and any reduction in viability of phage was recorded. Similarly, the effect of pH (3-11) was seen on viability of phages in broth.

#### Titration of phage lysate

Phage lysate was prepared as per protocol of Jothikumar *et al*. [8] with slight modification. Briefly, phage stock (100 µl) was mixed with 200 µl of respective host bacterial culture (3×10^7^ to 1×10^8^ cfu/ml). Then, 2.4 g/L of MgCl_2_ was added and kept for 20 min on shaker adjusted at a gentle speed. The suspension was then mixed into 2.5 ml molten soft agar kept at 45°C in water bath and incubated up to 48 h at 37°C. Confluently, lysed plates were selected and the top agar was scrapped with 5 ml of lactose broth. Scrapings were pooled and 2-3 drops of chloroform were added and kept for 10 min and then centrifuged at 5000 rpm for 20 min in a refrigerated centrifuge (Remi C-24). Supernatant was filtered through 0.45 µm Millipore syringe filter and filtrate was used as lysate. Ten-fold serial dilution of the phage lysate in normal saline was prepared and subjected to plaque formation by DAL method. Phage titer was expressed as plaque forming units (PFU/ml) and determined by following formula:

PFU/ml=Number of plaques × Dilution factor

## Results

### Phages isolates

Recovery of phages was slightly higher in dairy cattle farm waste as compared to buffalo farm waste, and concentration of phage was greater in bottom and middle layer than superficial layer of tank (Table 1). Lytic activity shown by turbidity reduction method was more (76.66%) as compared to streak plate method (55%) and DAL method (55%). Further, comparison of streak plate method with DAL method revealed 100% correlation.

**Table-1:** Recovery status of lytic phages.

Sources of sample	Strata of sampling	Number of samples	Lytic activity of phage by rapid screening method	

			Turbidity reduction method (%)	Streak plate method (%)
Dairy cattle farm	Top	14	6 (42.8)	6 (42.8)
	Middle	14	13 (92.8)	7 (50)
	Bottom	14	14 (100)	12 (85.7)
	Total	42	33 (78.57)	25 (59.62)
Buffalo farm	Top	6	3 (50)	0 (0)
	Middle	6	4 (66.7)	3 (50)
	Bottom	6	6 (100)	5 (83.3)
	Total	18	13 (72.22)	8 (44.44)

**Figure-1:**
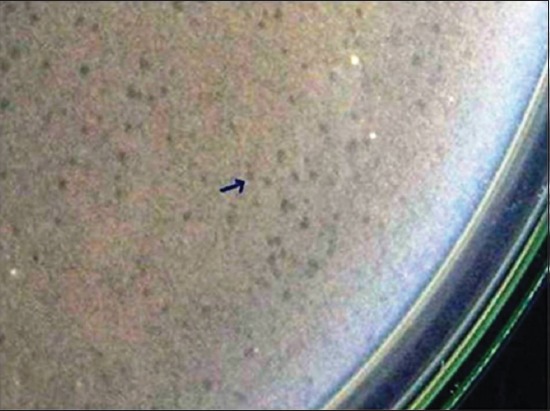
Small sized clear plaques on bacterial lawn (BsHR_1_).

**Figure-2:**
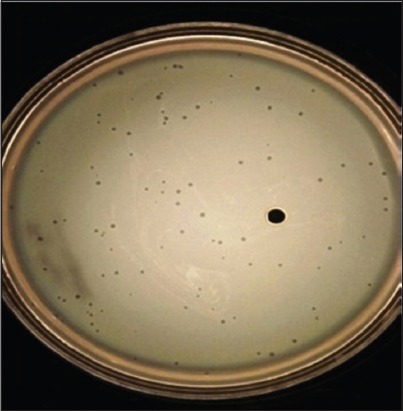
Small sized clear plaques on bacterial lawn (EHR_1_).

**Figure-3:**
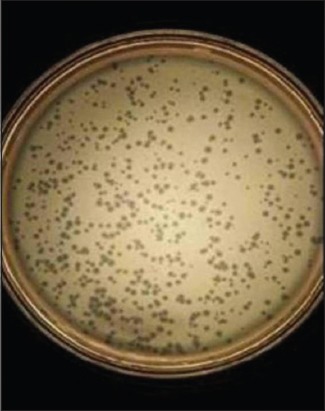
Large sized diffused plaques on bacterial lawn (EHR_2_).

**Figure-4:**
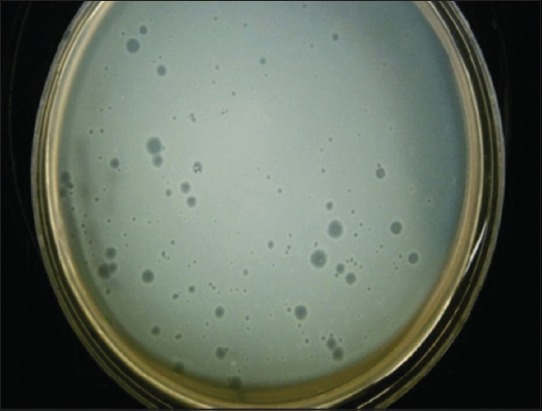
Elevated haloed plaques on bacterial lawn (EHR_3_).

During the present study, a total of 33 phage isolates were obtained. For each prime host, three different phage types were assigned on the basis of plaque morphology. Numbers of phage strains recovered against *B. subtilis* and *E. coli* were 30 (91%) and 3 (9%), respectively (Table 2). Three different phage morphotypes each against *B. subtilis* (BsHR_1_, BsHR_2_, and BsHR_3_) and *E. coli* (EHR1, EHR_2_, and EHR_3_) were reported (Figures-1-4).

**Table-2:** Plaque morphology of phage lysates.

Phage type	Number of isolates	Plaque morphology
BsHR_1_	22	Small sized pin headed, clear plaque
BsHR_2_	5	Small sized diffused/opaque plaque
BsHR_3_	3	Large sized clear plaque
EHR_1_	1	Small sized, clear plaque
EHR_2_	1	Large sized diffused plaques
EHR_3_	1	Elevated types haloed plaques

### Characteristics of phages

No significant effect due to pH on viability of phage isolates was recorded. The isolates appear to be stable at the pH range 5-9 but inactivation is evident at the very low (pH 3) and very high pH levels (pH 11). Phage isolates BsHR_2_ and BsHR_3_ remain viable at 70°C up to 3 min while BsHR_1_ showed a definite decrease in the percent survivability at 70°C for 2 min and no viability was seen when exposed for 3 min. However, EHR1, EHR2, and EHR3 isolates remained viable at 70°C up to 2 min with further inactivation when exposed for 3 min. Mean phage titer of lysate ranged from 3×10^12^ to 5×10^12^ PFU/ml.

### Host range of phage isolates

Lytic activity of phage lysate against other bacteria is illustrated in Table 3. BsHR_1_ and BsHR_2_ were lytic against *Staphylococcus*, *Salmonella*, and *Pseudomonas* while BsHR_3_ was found effective against only two genera, *viz*., *Staphylococcus* and *Pseudomonas*. Among the five genera tested none of the bacteria was found sensitive to EHR_3_. EHR_2_ showed lytic activity against *Staphylococcus* only while EHR_1_ had wide host range.

**Table-3:** Host range of phage isolates.

Phage type	Number of phage isolates	*S. aureus*	*Salmonella* spp.	*P. aeruginosa*	*Klebsiella* spp.	*Proteus* spp.
BsHR_1_	22	14	8	18	0	0
BsHR_2_	5	4	2	4	0	o
BsHR_3_	3	3	0	3	0	o
Total	30	21	10	25	0	0
EHR_1_	1	1	1	1	1	1
EHR_2_	1	1	0	0	0	0
EHR_3_	1	0	0	0	0	0
Total	3	2	1	1	1	1

S. aureus=Staphylococcus aureus, P. aeruginosa=Pseudomonas aeruginosa

## Discussion

Likewise present study, various workers have isolated phages from sewage of livestock farms [12,13]. In accordance with the previous study, rapid screening methods such as turbidity reduction and streak plate methods and subsequent processing by DAL method [12,14,15] were found much helpful during the present investigation. This highlighted the importance of initial screening for successful recovery of phage. Failure of streak plate method to give 100% recovery at par with that of turbidity reduction method may be attributed to less concentration of phages besides other factors, as large volume of sample is required for phage propagation. Those samples which failed to induce lysis of bacteria in turbidity reduction method also did not show plaque formation by streak plate method. Turbidity reduction method can be applied on crude sample and is comparatively convenient to perform and also is less time-consuming. However, the rapid turbidity reduction method alone was not found reliable as it frequently yielded false positive results also. Hence, simultaneous streak plate method that improved the recovery status of phage lysates is recommended. Most of the workers stressed on choice of appropriate host bacterium for optimum recovery of phages. There are several reports that support the use of either *B. subtilis* and/or *E. coli* [16,17]. *B. subtilis* supported the growth of multiple morphotypes of phages as compared to *E. coli* concluded on the basis of recovery of thirty *B. subtilis* (BsHR) phage and three *E. coli* specific phages (EHR). Similarly, Krasowska *et al*. [18] reported that phage had the highest percent adsorption to the *Bacillus* host. Due to high activity at the low and high temperature and pH, *B. subtilis* phage seems to be the best candidate for use in industry. Less recovery of *E. coli* phages may be correlated with the size of bacterium [13]. *E. coli* being smaller in size provides less surface area for attachment of bacteriophage as compared to *B. subtilis*.

Higher recovery status of phages in dairy farm waste as compared to buffalo farm waste is in support with report of Tiwari *et al*. [12], Shukla and Hirpurkar [13], and Askora *et al*. [19]. Higher concentration of phage in bottom and a middle layer of tank were also reported by Carey-Smith *et al*. [20] and Shukla and Hirpurkar [13]. Goyal *et al*. [21] opined that most of the organic matter settles in the deeper layer, thus providing optimum conditions for multiplication of host bacteria in deeper layer, which in turn, improves host range interaction.

Plaque morphology is one of the foremost criteria for characterization of phages [13]. Variation in the plaque morphology during the present study may correspond to the difference in phage strain, gel strength and addition of cation [12,22]. In contrast, Jothikumar *et al*. [8] found that plaque morphology was not affected by addition of cations. Pedroso and Martins [23] also did not find any relationship between coliphage family and specific plaque morphology.

Present findings are in accordance with earlier reports of Tiwari *et al*. [12] and Shukla and Hirpurkar [13] who observed that phage viability was maximal between pH 5 and 9 and all phages were completely inactivated at pH of 3 and 11. Likewise, Ibrahim *et al*. [24] observed stable lytic activities at pH 6-8. On the contrary, Krasowska *et al*. [18] reported *B. subtilis* phages resistant to the acidic (4.0) and alkaline (9.0 and 10.0) pH. Low pH reportedly affects phage aggregation and reduces their abilities to penetrate the host cells [25].

Temperature susceptibility of phages in present findings is in conformity to those reported by Tiwari *et al*. [12] and Shukla and Hirpurkar [13]. Likewise, Lu *et al*. [26] have also reported that phages get inactivated at 70°C and above, while our findings are contradictory to those reported by Krasowska *et al*. [18] who found that phages of *Bacillus* were resistant to high temperatures (80°C for 1 min). The reduction of burst size at higher temperature is probably the result of the effect of higher temperature on the metabolism of the host because the bacterial growth rate is decreased between 45°C and 51°C. Likewise present study, many other workers have obtained high titer phage lysate [26,27].

Wide host range and phage types reported during present study are in conformity to the reports of Bielke *et al*. [28] who observed that phage host range is not always genera restricted, so phages could have wide host range. Present observations are in partial conformity with Carey-Smith *et al*. [20] who had reported narrow range phages restricted to maximum of two bacterial species. During the last decade, a marked increase in the number of identified phages has been observed. More than 200 lytic *Staphylococcal* phages have been characterized [29]. Present findings are supported by earlier studies also in which phages were recorded against *Streptococci* [30], *E. coli* and other enterobacteria [31], *Pseudomonas* [32], *S. aureus* [33], and *Bacillus* [16].

## Conclusion

This study isolated and characterized phages from dairy farm waste disposal using *E. coli* and *B. subtilis* as the host system. Recovered phage lysate had broad host range displaying their potential to be used as therapeutics in infectious diseases. However, there is need to understand *in vivo* phage-mediated selection.

## Authors’ Contributions

RKS and SDH designed the experiment. Sample collection was done by RKS and VP. Media preparation and laboratory analysis were performed by RKS, CS, and NR under the supervision of SDH. All authors read and approved the final manuscript.
